# Effects of adults’ health behaviors and combinations thereof on health outcomes: an analysis using National Health Insurance Service of Korea cohort data

**DOI:** 10.4178/epih.e2019042

**Published:** 2019-10-08

**Authors:** Hyun-Jung Park, Eun-Jung Kim

**Affiliations:** Department of Nursing, Pyeongtaek University, Pyeongtaek, Korea

**Keywords:** Health behavior, Outcome assessment, Chronic disease, Obesity, National Health Insurance Service, Korea

## Abstract

**OBJECTIVES:**

The purpose of this study was to estimate the effects of health-risk behaviors, alone and in combination, on health outcomes.

**METHODS:**

This study used sample cohort data provided by the National Health Insurance Service focusing on the use of hospital services, direct medical expenses, length of stay, and re-entry rate according to health-risk behaviors. A frequency analysis and the chi-square test were used to investigate associations between the demographic characteristics of study subjects and their health-risk behaviors. The strength of the association of each factor was calculated as the odds ratio in a crossover analysis.

**RESULTS:**

Obesity had the largest effect, especially in combination with smoking and drinking. In particular, significant associations were shown with the duration of hospitalization and direct medical expenses. After adjustment for sex, age, economic status, and pre-existing medical conditions, the duration of hospitalization was 7.37 times longer and that of medical expenses was 5.18 times higher in the obese group relative to the non-obese group. Drinking showed a statistically significant association with the number of days of hospitalization. After adjusting for the control variables, the number of hospital days was 1.24 longer in the drinking group than in the non-drinking group.

**CONCLUSIONS:**

An analysis of combinations of health risk factors showed obesity had the largest effect.

## INTRODUCTION

Expenditures for health insurance and medical expenses due to smoking, drinking, and obesity have steadily increased, and expenditures for health insurance and medical expenses due to smoking increased by 48.7% from 1.5120 trillion Korean won (KRW) in 2007 to 1.5633 trillion KRW in 2011. The costs associated with alcohol consumption increased over the same period by 42.7%, from 1.705 trillion KRW to 2.4336 trillion KRW, while those related to obesity increased by 41.9%, from 1.8971 trillion KRW to 2.6900 trillion KRW [1]. Unhealthy behaviors such as smoking, drinking, and obesity increase the prevalence of chronic diseases such as cancer, diabetes, and cardiovascular disease throughout the world [2], consequently increasing medical expenses. In addition, such behaviors cause enormous health-related costs related to disability, shortened life expectancy, and reduced productivity [3]. According to a study by the World Health Organization, 7% of deaths worldwide are due to non-communicable or chronic diseases [4]. Smoking is associated with 71% of lung cancer deaths and 18% of total deaths. Drinking is associated with 6% of total deaths, 7% of reduced life expectancy, 44% of cases of diabetes, and 23% of cases of cardiovascular disease. It is known that 7-41% of cancer cases are caused by obesity [4]. A previous study developed a theoretical model for health-risk behaviors, and empirical studies have been published on the effects of health-risk behaviors, as well as their causes, consequences, and policies to reduce them [5]. In addition, Grossman [6] stated that health behaviors (cessation of smoking or alcohol, or an increase in exercise, etc.) persist until the marginal cost equals the marginal income, based on the health resource model. Strum analyzed the effects of obesity, smoking, and high-risk drinking on health service utilization and health status [7], and explored the effects of smoking, drinking, and obesity on the frequency of medical visits for primary care [8]. That study emphasized the necessity of an intervention program for smoking, which was the most important factor contributing to increased medical expenses. Vals et al. [9] analyzed the effects of smoking, drinking, and obesity on health-related services and outcomes in Estonia.

However, it is known that health-risk behaviors exist in clusters. That is, each health-risk behavior does not exist independently, but is closely correlated with other behaviors [10]. Smoking and frequent drinking are associated with low fruit and vegetable intake [11], and some studies have analyzed the relationship between smoking and drinking [12]. The 10-year survival probability of elderly people over 65 years of age who had several health-risk behaviors was significantly lower than that of their counterparts who did not, and studies have statistically demonstrated associations between health-risk behaviors and heart disease [13]. Lin [14] conducted a study to examine the relationship between health risk factors and health care expenditures among people aged 12 years or older in Taiwan. For outpatients, the cost of treating ex-smokers was 32% higher than that of treating current smokers and non-smokers. Meanwhile, the cost of treating daily alcohol consumption was 29% higher than that of treating non-daily drinkers, and the cost of treating those who exercised frequently was 9% lower than that of treating those who exercised infrequently. For inpatients, past smoking and physical activity (PA) were found to have significant positive and negative relationships with medical expenditures, respectively [14]. Musich et al. [15] used health claims data to characterize the relationship between health risk factors and medical costs for hospitalization and supplementary treatment. The high-cost group, which was defined as the top 10% of health expenditures for the top 10% of the health risk group, showed 1.70 times higher costs than their counterparts. The worse the health behavior, the higher the level of medical expenditures. In addition, studies that evaluated the validity and reliability of low exercise frequency as a risk factor found it to have a significant relationship with expenditures, and was clustered with high-risk drinking and smoking [16].

The purpose of this study was to estimate the effect of health-risk behaviors on health outcomes from a multidimensional vantage point. For this purpose, sample cohort data of the National Health Insurance Service (NHIS) were used, focusing on the use of hospitalized services, direct medical expenses, length of stay, and re-entry rate according to health-risk behaviors. Improving health outcomes not only means treating illnesses, but also includes improving overall quality of life and promoting psychological stability. Therefore, health outcomes need to be analyzed to promote healthy lives. In addition, sample cohort data are highly valuable for biomedical research. Cohort subjects are representative of the entire population and cohorts include information on disease and mortality, as well as demographic and socioeconomic information. Data from national health examinations also include basic test results, information on health behaviors, and physical measurements. The re-collection of the subjects’ data is also a major advantage, since the effects of factors that change over time can be considered. Therefore, this source provided representative data for the analysis presented herein.

## MATERIALS AND METHODS

### Data

In this study, we used insurance qualification data, medical data, health examination data, and medical institution data. Since health-risk behavior variables are used in health screenings, the qualification data and the medical data could be merged to link the cost of medical care, length of stay, and re-admission.

To assess lifestyle factors, lifetime smoking was used as a variable, as was alcohol consumption per week. High-risk drinking was defined as drinking 7 or more glass in two times a week for males and drinking 5 or more glass in two times in a week for females [17]. Obesity was defined in terms of body mass index (BMI; kg/m^2^) based on measurements of weight and height. Overweight and obesity were defined as dummy variables, using a BMI threshold of 25.0 kg/m^2^.

To measure health outcomes, the number of days of hospitalization, direct medical expenses, and re-admission were used as variables. These outcomes were chosen due to data availability. The number of days of hospitalization was defined as the average number of days that a subject was hospitalized, from the time of admission to discharge, between 2011 and 2013. This value was transformed using the natural logarithm because it was not normally distributed. For medical expenses, the amount borne by health insurance benefits was excluded. We instead used the average annual statutory copayment, which included emergency medical expenses, inpatient medical expenses, outpatient medical expenses, and medication costs. Re-admissions were defined as the same subject being re-admitted due to the same disease (primary diagnosis using International Classification of Diseases, Tenth Revision codes) and re-admitted during the study period.

In addition, we used sex and age as predecessor factors, with demographic variables included as exogenous variables. Economic status was selected as a control variable. To control for health status, we included the 3-year average of the number of deaths caused by stroke, heart disease, hypertensive disease, diabetes, hyperlipidemia, and pulmonary tuberculosis as a risk factor. These 6 diseases are non-health behavior–related comorbidities. It was necessary to consider previous health status in order to avoid biasing the health outcomes according to comorbidities.

### Data analysis

A frequency analysis and the chi-square test were used to investigate the relationship of the demographic characteristics of the study subjects with their health risk behaviors and combinations thereof. The strength of the association between each factor was calculated as the beta coefficient and odds ratio (OR) in a cross-over analysis. The association between health behavior combinations and health outcomes was explored using logistic regression after adjusting for sex, age, economic status, and the number of comorbidities. We used combinations of health behaviors as independent variables and health outcomes as dependent variables. The reference group was those who did not engage in any of the 3 health-risk behaviors. SPSS version 21.0 (IBM Corp., Armonk, NY, USA) was used for the statistical analysis.

### Population

The total number of observations for the 2011-2013 examination period was 680,300. Subjects who were under 20 years of age (n=1,431), Medical Aid recipients (n=6,185), and underweight (n=25,003) were excluded, as were those with missing health risk variables (n=3,119). The final sample totaled 56,221 people. Medical Aid recipients were excluded because of differences in their medical institution utilization pattern and medical cost scale, and underweight subjects were excluded because the prevalence of other diseases (not related to obesity, smoking, or drinking) is high in this group, potentially affecting their health outcomes. Since BMI was utilized as an indicator of obesity, underweight individuals were excluded using BMI as a criterion [18]. The remaining 56,221 individuals in the cohort were observed for 3-year and selected as the final subjects. In this analysis, 3-year average values of data for smoking, drinking, and BMI were used. Since the medical checkup items were changed in 2009 and the identification codes linked to drug expenditures were made available starting in 2011, the analysis was conducted using data from 2011 to 2013.

### Ethics statement

The use of these data was approved by the NHIS Ethics Committee (NHIS-2016-2-253).

## RESULTS

The general characteristics of the subjects are shown in Table 1. Of the subjects, 42.8% were males and 57.2% were females, with an average age of 50.30 years. The average income quintile was 2.24, and the number of pre-existing diseases was 2.08 on average. The smokers had smoked 14.08 cigarettes a day for 19.64 years on average and the drinkers consumed 4.57 drinks of alcohol per drinking session. The mean BMI was 24.91 kg/m^2^, and 32.9% of the subjects were overweight and obese. Overall, the subjects had 1.74 health-risk behaviors on average. The average number of hospitalized days was 13.80 days per year, and roughly 250,000 KRW was spent on medical expenses. Approximately 16.9% of the patients (n=56,221) who were hospitalized experienced re-admission.

Table 2 shows the results for the various health outcomes according to the frequency of health-risk behaviors. For those who did not exhibit any health-risk behaviors, the average duration of hospitalization was 5.1 days. Their medical expenses were about 25,000 KRW, and their re-admission rate was 4%. For subjects with 1 health-risk behavior, smokers had the longest average hospitalization (approximately 13 days). However, those with obesity had the highest direct medical costs (99,000 KRW) and the highest re-admission rate. In the analysis of subjects belonging to 2 or more health risk groups, the highest duration of hospitalization was found for subjects with smoking and obesity as combined risk factors. The average value of health expenditures of this group was 141,547 KRW, and roughly 16.9% experienced re-admission (Table 1). Those with obesity and drinking as risk factors had expenditures of 152,140 KRW with an average hospitalization duration of roughly 21 days. Finally, subjects with all 3 health-risk behaviors, the average hospitalization duration was 25 days, approximately 260,000 KRW was spent related to hospitalizations, and 24% of the subjects experienced re-admission.

Table 3 shows the relationships of health outcomes with health-risk behavior clusters. In subjects with 1 risky behavior, there was no statistically significant association between smoking and any health outcomes, while obesity was found to have significant relationships with the number of hospital days and direct medical expenses. After adjusting for sex, age, economic status, and the number of existing diseases, the obese group was 7.37 times more likely to be hospitalized than the non-obese group and spent 5.18 times more on medical expenses. A significant association was found between drinking and the number of days of hospitalization. After adjusting for the control variables, the number of days of hospitalization was found to be 1.24 times longer than the non-drinking group.

For subjects with smoking and obesity as health-risk behaviors, the number of days of hospitalization was 3.11 times longer and the direct medical costs were 1.97 times higher than those for subjects who exhibited 1 health-risk behavior and those who did not exhibit any health-risk behaviors, respectively. For those with both smoking and drinking as health-risk behaviors, direct medical costs were 1.19 times higher. Finally, the presence of both obesity and drinking significantly affected all health outcomes. The number of admission days increased by about 1.24 times, the direct medical costs increased by 2.04 times, and the re-admission rate was 1.99 times higher. Among the subjects who belonged to all health-risk groups, the number of hospitalization days was 2.21 times longer, and medical costs were 4.44 times.

## DISCUSSION

There is a growing interest in health outcomes, as well as measures of the prevalence or incidence of chronic diseases. Health outcomes are indicators of clinical outcomes, health-related quality of life, and satisfaction with treatment, which can be classified into outcomes. Clinical outcomes include patients’ signs and symptoms, laboratory results, and mortality, while health-related quality of life refers to patients’ health status, including their level of mental, physical, and social wellness. Meanwhile, medical satisfaction refers to the accessibility, convenience, and quality of care [19]. In other words, improving health outcomes not only means treating illnesses, but also includes improving overall quality of life and promoting psychological stability [20].

The data used in this study consisted of the sample cohort data provided by the NHIS (National Health Insurance Service National Sample Cohort 2002-2013), which consist of qualification data, medical data, health examination data, and medical institution data. In this study, the health-risk behavior variables used as the main variables were identified and constructed from the health screening data, and the qualification data and the medical data were merged to identify the demographic variables, medical expenses, length of stay, and re-admission rate according to individuals’ identification number.

Differences in health outcomes were found according to individual health-risk behaviors and their combinations. The presence of smoking as the sole health-risk behavior showed no statistically significant association with any health outcomes, while obesity as the only health-risk behavior had significant relationships with the number of hospital days and direct medical expenses. After adjusting for sex, age, economic status, and existing disease, the obese group had 7.37 times more hospitalized days and spent 5.18 times more on medical costs than the non-obese group. Drinking as the sole health-risk behavior showed a significant relationship with the number of hospital days. After adjusting for the control variables, the number of hospitalized days was 1.24 times higher in the drinking group than in the non-drinking group. Obesity showed the greatest impact on health outcomes; in particular, it had a greater impact on the number of hospitalized days than on the cost of health care. These results are consistent with various existing research findings.

A previous study found higher levels of binge drinking and stress were associated with a higher likelihood of medical treatment [21]. Another study reported that men did not show any relationship between PA and medical expenses related to obesity, but females who engaged in PA had 0.8 times lower medical expenses [22]. Another study analyzed the effect of smoking cessation on obesity [23]. As age increases up to a certain point, the likelihood of obesity increases, and then subsequently decreases. Rice et al. [24] found a high frequency of alcohol consumption in their study of drinking behavior and the use of health service utilization. Furthermore, consuming more than 6 drinks a day was associated with a 1.13 times higher likelihood of receiving care at a medical institution than consuming fewer than 2 drinks per day [25]. Cha & Yoon [26] compared the health behaviors and medical costs of patients with diabetes according to whether they engaged in exercise. After controlling for socioeconomic variables, it was reported that the medical expenditures of patients who did not engage in exercise were higher. Another study investigated the relationship between health behaviors and health care expenditures among participants in the Health Enforcement Research Organization. The factors affecting the top 10% of health care expenditures for smoking were past smoking history, current smoking, and PA, which were associated with 19.7%, 14.5%, and 10.3% higher medical expenditures, respectively [27]. Only 42% of males and 63% of females had a single risk factor; more specifically, the rate of the lack of PA was high among females [28]. This is consistent with our findings.

For subjects with 2 or more health risks, the number of days of hospitalization was 3.11 times higher and direct medical expenses were 1.97 times among those with smoking and obesity as risk factors, and direct medical expenditures were about 1.19 times higher in those with smoking and drinking as risk factors. The combination of obesity and drinking significantly affected all health outcomes. The number of hospitalization days was 1.24 times higher, direct medical expenses were 2.04 times higher, and the re-admission rate was 1.99 times higher in this group. Among subjects with smoking, obesity, and drinking as risk factors, the number of hospitalization days was 2.21 times higher, and medical costs were 4.44 times higher than in the reference group. For a cluster of 2 or more risk factors to be significant, the ratio of the observed frequency and the expected frequency should be similar to that reported in a study in which all clusters were significant in males [11]. As previous studies have shown that smoking and drinking are closely correlated, smoking and high-risk drinking are assumed to be common links to other lifestyle habits [29-31]. All health-risk behaviors should be improved simultaneously. However, a previous study has suggested that obesity should be classified as a health-risk behavior with unique characteristics [30]. That study also concluded that the health outcomes of individuals with both smoking and obesity or both alcohol and obesity as risk factors were worse than those of individuals with only smoking or drinking as health-risk behaviors. Therefore, it is necessary to carry out research into multi-collinear interactions among health hazards using clinical data.

The results of this study are based on an investigation into combinations of health-risk behaviors and their relationships with hospitalization days, medical costs, and the re-admission rate. The effect of obesity was highest when it was accompanied with other health-risk behaviors; in addition, its effect was stronger than those of smoking and drinking as single health-risk behaviors.

However, caution is needed when interpreting the results of this study. This study separated health-risk behaviors and simplified their characteristics. Nonetheless, the correlation between smoking and drinking is high, and the relationship between drinking and obesity cannot be ignored. Therefore, an arithmetic analysis is needed to correct this. In addition, this study used only the presence of existing diseases as a control variable. However, the severity of stroke, heart disease, hypertension, diabetes, hyperlipidemia, and pulmonary disease may have distinct effects on health-risk behaviors. Unfortunately, due to limitations of the data, these effects could not be reflected. Therefore, caution is needed in interpreting these findings due to uncollected data. Nevertheless, the present study quantitatively assessed the risk of multiple risk behaviors and obesity instead of single health-risk behaviors. In addition, the statistical confirmation that these health risk behaviors impact length of hospitalization more than the re-admission rate is a significant finding. Another strength of this study is its use of the cohort database, which is a large database that can be used verify the effects of long-term prospective cohort studies or interventions [32]. Korea’s national health insurance system, which covers the entire population, is recognized globally. The NHIS Sample Cohort data are constructed through a stratified system extraction method based on individuals’ medical history, medical examination results, residence, insurance premiums, and information on nursing institutions; therefore, it can be considered a representative data source for national health information.

Based on this study, it is necessary to review chronic disease management policies to approach and manage the health risks caused by smoking, drinking, and obesity, and to implement an integrated approach to chronic disease management.

## Figures and Tables

**Table 1.** General characteristics of the subjects (n=56,221)

Variables	n (%) or mean±SD
Sex	
Female	32,176 (57.2)
Male	24,045 (42.8)
Average age	50.30±0.46
Income quintile	2.24±1.88
Existing diseases^1^	2.08±0.12
Smoking	
Cigarettes per day	14.08±0.44
Smoking duration (yr)	19.64±9.68
Total pack-years	107.22±98.57
Alcohol consumption	
Drinks in a single sitting (glasses)	4.57±3.29
Frequency per week	1.01±1.87
Amount per week	4.97±12.99
Obesity	
Average BMI	24.91±2.69
Obese subjects (BMI ≥25.0 kg/m^2^)	18,483.00±32.88
No. of health-risk behaviors	1.74±1.01
Admission days (d/yr)	13.80±21.33
Direct medical costs (Korean won)	247,987.00±359,114.01
Re-admission	
Yes	9,479 (16.9)
No	46,742 (83.1)

SD, standard deviation; BMI, body mass index.

1 Stroke, cardiovascular diseases, hypertensive disease, diabetes mellitus, hyperlipidemia, tuberculosis.

**Table 2.** Average values of health outcomes according to health-risk behaviors

No. of health-risk behaviors	Smoking	Obesity	Drinking	Admission (d)	Direct medical costs (KRW)	Re-admission^1^
0	-	-	-	5.07±1.28	24,887.24±13,258.21	0.04±0.24
1	+	-	-	12.75±8.21	75,410.64±62,557.12	0.08±0.09
	-	+	-	7.87±1.99	98,776.22±42,198.77	0.10±0.04
	-	-	+	7.94±2.04	64,137.18±43,947.19	0.06±0.17
2	+	+	-	21.79±14.87	141,547.07±54,971.11	0.21±0.25
	+	-	+	19.01±5.48	115,497.23±51,317.54	0.19±0.63
	-	+	+	20.54±11.10	152,140.72±95,847.59	0.18±0.11
3	+	+	+	25.11±9.21	259,140.11±85,547.11	0.24±0.65

Values are presented as mean±standard deviation. +: health-risk behavior present; -: health-risk behavior absent.

1 Experienced=1, none=0.

**Table 3.** Health outcomes according to health-risk behaviors^1^

Health-risk behavior clusters		Admission days			Direct medical costs			Re-admission		
		Beta	95% CI		Beta	95% CI		OR	95% CI	
			LL	UL		LL	UL		LL	UL
Smoking	Before adjustment	0.97	0.53	1.95	1.01	0.88	1.15	1.21	0.97	1.42
	After adjustment	0.67	0.55	1.25	0.94	0.62	1.91	1.08	0.74	1.21
Obesity	Before adjustment	7.37^**^	5.57	16.27	5.18^**^	4.58	9.31	2.11	0.94	3.08
	After adjustment	6.21^***^	5.99	12.43	4.44^**^	3.11	14.25	1.64	0.57	5.79
Drinking	Before adjustment	1.97	0.54	2.57	2.07^**^	1.43	3.27	2.07	0.57	6.87
	After adjustment	1.24^**^	1.02	1.89	1.93	0.14	3.58	1.55	0.33	2.01
Smoking and obesity	Before adjustment	5.02^**^	1.27	9.25	2.52^**^	1.38	5.27	0.97	0.14	3.22
	After adjustment	3.11^**^	2.10	5.96	1.97^**^	1.11	3.27	0.54	0.27	1.94
Smoking and drinking	Before adjustment	1.24	0.82	1.36	1.24	0.71	1.67	2.11	0.67	3.00
	After adjustment	1.14	0.57	5.15	1.19^**^	1.12	1.54	1.95	0.17	4.18
Obesity and drinking	Before adjustment	1.52^**^	1.02	2.07	1.98	0.11	2.57	2.17^**^	1.96	2.57
	After adjustment	1.24^**^	1.01	1.47	2.04^**^	2.00	3.11	1.99^**^	1.53	2.03
Smoking, obesity, and drinking	Before adjustment	5.38	0.49	5.44	6.24^**^	2.87	7.18	2.11^*^	1.94	3.07
	After adjustment	2.21^**^	2.01	2.48	4.44^**^	2.87	5.11	1.76	0.27	1.97

Adjusted for sex, age, economic status, and number of previous diseases. OR, odds ratio; CI, confidence interval; LL, lower limit; UL, upper limit.

1 Reference group: all 3 health-risk behaviors absent.

* p<0.05,

** p<0.01,

*** p<0.001.
